# Cardiovascular and Pre-Frailty Risk Assessment during Shelter-In-Place Measures Based on Multimodal Biomarkers Collected from Smart Telemedical Wearables

**DOI:** 10.3390/jcm10091997

**Published:** 2021-05-06

**Authors:** Eliasz Kańtoch, Anna Kańtoch

**Affiliations:** 1 AGH University of Science and Technology, 30-059 Krakow, Poland; 2 Jagiellonian University Medical College, Faculty of Medicine, Department of Internal Medicine and Gerontology, 30-688 Krakow, Poland; anna.kantoch@doctoral.uj.edu.pl

**Keywords:** frailty, telemedicine, gerontechnology, wearable sensors, shelter-in-place measures, Covid-19, resting heart rate, sleep duration, activity pattern

## Abstract

Wearable devices play a growing role in healthcare applications and disease prevention. We conducted a retrospective study to assess cardiovascular and pre-frailty risk during the Covid-19 shelter-in-place measures on human activity patterns based on multimodal biomarkers collected from smartwatch sensors. For methodology validation we enrolled five adult participants (age range: 32 to 84 years; mean 57 ± 22.38; BMI: 27.80 ± 2.95 kg/m^2^) categorized by age who were smartwatch users and self-isolating at home during the Covid-19 pandemic. Resting heart rate, daily steps, and minutes asleep were recorded using smartwatch sensors. Overall, we created a dataset of 464 days of continuous measurement that included 50 days of self-isolation at home during the Covid-19 pandemic. Student’s t-test was used to determine significant differences between the pre-Covid-19 and Covid-19 periods. Our findings suggest that there was a significant decrease in the number of daily steps (−57.21%; −4321; 95% CI, 3722 to 4920) and resting heart rate (−4.81%; −3.04; 95% CI, 2.59 to 3.51) during the period of self−isolation compared to the time before lockdown. We found that there was a significant decrease in the number of minutes asleep (−13.48%; −57.91; 95% CI, 16.33 to 99.49) among older adults. Finally, cardiovascular and pre-frailty risk scores were calculated based on biomarkers and evaluated from the clinical perspective.

## 1. Introduction

Coronavirus disease 2019 (Covid-19) lockdown polices were introduced worldwide during a global health crisis to combat the spread of the novel coronavirus (Severe Acute Respiratory Syndrome Coronavirus 2—SARS-CoV-2) pandemic, forcing people to change their behavior, daily habits, and routines which affected their activity level. However, the quantitative impact of these measures on older adults is unknown. Keesara et al. [1] reported that to face this crisis, we need an immediate digital revolution and the transformation of health care delivery into some telemedical solutions to protect against the spread of the pathogen to uninfected patients in a clinical and non-clinical settings. The Covid-19 pandemic has accelerated the paradigm shift in healthcare from traditional care to telemedical care. However, the procedures and methods for supporting healthcare professionals are not clearly defined, as well as there are challenges related to telemedical technologies. In 2019, Sim reported that major challenges for mobile health include the discovery and validation of digital biomarkers, and the regulation of mobile health technologies [2].

Interestingly, the Covid-19 pandemic has already disrupted health care and has accelerated the development of telemedical services. Over the past decade, many telemedical services are gaining traction, including teleconsultations, e-prescriptions, teleradiology, telecardiology. However, Keesara et al. reported that telemedical digital technologies had low penetration into the market [1].

During the pandemic, we observe a rapid shift from in-person doctor visits to teleconsultation based on information and telecommunication technologies, which becomes challenging for both patients and doctors. Teleconsultation, however, has several drawbacks and limitations. The major limitation is the lack of measured health parameters, which are often performed during a medical visit, such as auscultation, heart rate measurement, body temperature, etc., and the diagnosis is mainly based on the patient’s medical interview.

Widespread mobile health technologies, especially wearable devices (e.g., smartphones, fitness trackers) may play a crucial role in the transition process and fill the missing category of home-based monitoring medical devices. We believe that data from wearable devices (such as smartwatches or fitness trackers) can be an auxiliary tool to assist teleconsultation with primary care physicians, especially in emergencies or health crises, because they may collect health data in the home setting, which can play an essential role in patient diagnosis or screening. Our previous work focused on investigating the desirable features and applications of telemedical services for the older adults delivered by wearable medical devices [3]. At present, smartwatches and fitness trackers are used to quantify physical activity and sleep quality with the primary goal of improving overall health. However, novel emerging applications include motion analysis and biomechanics, rehabilitation, active assistive living, and health parameters monitoring. Furthermore, wrist-worn activity trackers are now a validated tool to assess physical activity in chronic diseases such as atrial fibrillation [4].

Frailty syndrome is associated with a high incidence of adverse health outcomes in the geriatric population, including an increased risk of hospitalization, falls, disability, institutionalization, and mortality. Frailty can be reversed either spontaneously or through nutritional interventions and exercise [5]. Screening for frailty syndrome is recommended to identify older adults who would benefit most from a Comprehensive Geriatric Assessment [6]. There are many scales and questionnaires for assessing frailty syndrome. However, the most common is the frailty phenotype, also known as Linda Fried’s Criteria, which includes five of the following criteria: exhaustion, unintentional weight loss, low physical activity, muscle weakness (handgrip strength), and slow gait speed. Frailty is defined as a clinical syndrome with at least three criteria, while pre-frailty is defined as the presence of one or two of these criteria [7]. It is estimated that the frailty syndrome affects 7–16.3% of the population aged 65 and over, and 25–40% of those aged ≥80 years, and the risk of frailty increases with age. Moreover, it is twice as high in women as in men [5,7,8,9]. Therefore, in our study, we tried to focus on octogenarians to create a useful prediction tool for Cardiovascular and Pre-Frailty Risk Assessment, as people 80 years of age and older are at increased risk of not only cardiovascular adverse events but also frailty due to their age.

The state of knowledge about the impact of Covid-19 restrictions on human activity is limited and is based mainly on online surveys. Recently, Gjaka et al. [10] published comprehensive research in which they investigated the effects of Covid-19 restrictions on physical activity based on online surveys. The authors showed that the restrictions had a negative impact on physical activity. It would be interesting how those results compare to the ground truth measured by movement sensors. Aggregate data from sports wristbands manufacturers are also available. Unfortunately, it is often difficult to analyze them because methodology is not clearly described and there is no information about population, data quality and consistency. Furthermore, the timetable for introducing the Covid-19 restrictions varied across countries and regions, which made data analysis difficult.

The aim of this paper was to propose a non-invasive triage methodology for the assessment of the cardiovascular and pre-frailty risk based on multimodal biomarkers collected from smartwatch sensors. For methodology validation we enrolled five adult participants (age range: 32 to 84 years; mean 57 ± 22.38; BMI: 27.80 ± 2.95 kg/m^2^) categorized by age who were smartwatch users and self-isolating at home during the Covid-19 pandemic. Resting heart rate, daily steps, and minutes asleep were recorded using smartwatch sensors. Overall, we created a dataset of 5.68 person-years (464 days) of continuous measurement and clinical follow-up by a geriatric specialist that included 50 days of self-isolation at home during the Covid-19 pandemic. The novelty of research is twofold: first, the quantitative impact of shelter-in-place measures on biomarkers was investigated in two age groups and mapped to the cardiovascular and pre-frailty risk scale, secondly a non-invasive method to assess the cardiovascular and pre-frailty risk has been developed and validated during shelter-in-place measure in collaboration with a geriatric specialist to screen vulnerable patients. The significant advantage of this approach is the non-invasive measurement, continuous assessment, quantitative scale, and a telemedical interface. Study results might impact the clinical practice by providing easy to deploy, low-cost, and scalable tool for assessing cardiovascular and pre-frailty risk. In addition, the paper discusses the advantages of wearable telemedical devices as a future physician tool to screen the patient’s overall health and improve the telehealth visit experience, especially during emergency scenarios (i.e., a pandemic).

We took advantage of global Covid-19 pandemic shelter-in-place measures to find a new perspective on human behavior during a pandemic. It is believed that people at home perform less physical activity, and their lifestyle is relatively sedentary, so the risk of frailty syndrome [11] and cardiovascular disease (CVD) increases [12]. Cardiovascular disease is a leading cause of death and many adverse outcomes among the elderly, including morbidity, disability, and the risk of hospitalization [13,14]. According to the 2016 European Guidelines on Cardiovascular Disease Prevention in Clinical Practice, healthy adults of all ages should exercise at least 150 min a week of moderate-intensity or 75 min of vigorous activity a week or in equivalent combinations [12]. What is more, the risk of frailty was significantly lower in older adults who walk at least 5000 steps a day [15].

However, during a period of self-isolation, these goals could be difficult to achieve due to limited possibilities of physical activity at home and a lack of motivation [16]. One of the solutions may be gamification, which may have a positive impact on health, wellbeing and motivation to exercise through interactive training programs [17]. Exergames were used in the population of long-term care residents to improve mobility [18]. Moreover, video games turned out to be an exciting option for motivating patients in the rehabilitation process [19].

We have organized the rest of this paper as follows. Section 2 is based on medical experience and provides an overview of the most critical parameters that can be measured with a smartwatch, and that can be useful during a medical teleconsultation. Section 3 describes the developed method to assess cardiovascular and pre-frailty risk and material. Section 4 presents the results. Section 5 discusses the results in the context of the Covid-19 pandemic. Finally, Section 6 concludes the article.

## 2. Clinical Point of View

One of the main risk factors for cardiovascular diseases is a sedentary lifestyle. It is believed that a sedentary lifestyle could easily be verified using a fitness tracker. However, physical activity, which could also be monitored by this device, has a positive effect on the frailty syndrome and many cardiovascular risk factors such as hypertension, cholesterol level, body weight and type 2 diabetes mellitus. What is more, regular physical activity could not only improve fitness and mental health but also reduce the risk of many adverse health outcomes [12].

### 2.1. Resting Heart Rate

Heart rate is the most common vital sign measured in clinical practice to assess cardiovascular health because it could be an excellent indicator of myocardial metabolism and cardiac output. The diagnosis of tachycardia, defined as an atrial and/or ventricular rate of >100 beats per minute (bpm), could be very dangerous because it could lead to many adverse events, including cardiomyopathy, myocardial ischemia, hypotension, low cardiac output, cardiac arrest, or even death [20].

Resting heart rate (RHR) is a determinant of prognostic value, indicating that elevated RHR values (RHR > 80 bpm) were associated with an increased incidence of cardiovascular disease and all-cause mortality compared to RHR < 60 bpm [21,22,23,24]. What is more, high RHRs were related to CVD complications and mortality among patients with type 2 diabetes mellitus [21]. Lower levels of RHR could be achieved through regular physical activity, which acts through the autonomic nervous system [21].

### 2.2. Sleep Duration and Quality

Sleep is a biological process that plays a crucial role in brain function and physiology, including metabolism, appetite regulation, and cardiovascular, hormonal, and immunological functions. According to the American Academy of Sleep Medicine classification, sleep could be divided into rapid eye movement (REM) and non-rapid eye movement (NREM) phases. NREM sleep consists of three stages: lighter (stages N1 and N2) and deeper sleep (stage N3) [25]. Sleep duration and quality are significant measures of sleep [26]. It is difficult to assess the perfect amount of sleep, which could be applied by everyone [27]. However, prolonged sleep time (>8 h/day) or shorter sleep times (<7 h/day) could be associated with many adverse health outcomes, including CVD, hypertension, type 2 diabetes, obesity, depression, and all-cause mortality [27,28,29]. Sleep quality is difficult to define and measure objectively, but it could be affected by many factors, such as environmental, psychological, or lifestyle behaviors: use of alcohol, caffeine, or stimulants, and use of phones or computers before sleep time [30,31]. Furthermore, poor quality of sleep could be associated with various adverse health outcomes [32].

### 2.3. Number of Daily Steps

A fitness tracker, which includes a pedometer, is a useful device to monitor the number of daily steps and provide feedback to promote walking [33]. Although 10,000 daily steps are widely recommended, evidence from previous prospective studies is still incomplete. According to the literature, a greater number of steps was associated with lower CVD, cancer, and all-cause mortality. Furthermore, a higher gait speed could also be associated with a reduced risk of death [34].

### 2.4. A Wearable Device for Health Tracking

Wearable devices are becoming more and more important in health tracking. A new category of medical wearable devices has emerged. Much recent research has shown that smartwatches can be widely used in healthcare. Smartwatches are used for monitoring activity or gait, chronic disease self-management, and nursing or home-based care. A systematic review by King et al. found that most studies used smartwatches to monitor Parkinson’s disease or epilepsy [35]. Other possibilities of using smartwatches have found application in diabetes, dementia, asthma, and stroke, or in people with visual impairment [35,36]. Our previous study focused on assessing cardiovascular risk using machine learning based on the developed T-shirt equipped with wearable sensors [37]. Recent research indicates that smartwatches can detect Covid-19 before symptoms appear.

## 3. Materials and Methods

### 3.1. Cardiovascular and Pre-Frailty Risk Assessment (CFRA)

We developed a 4-point Cardiovascular and Pre-Frailty Risk Assessment (CFRA) method that provides a quantitative method for assessment of the cardiovascular and pre-frailty risk. We chose the following biomarkers: daily steps, resting heart rate, sleep duration, and age. The more points scored, the higher probability of developing cardiovascular health outcomes and frailty syndrome. The Cardiovascular and Pre-Frailty Risk Assessment 4-point method was exhibited in Table 1.

### 3.2. Study Design

We conducted a retrospective study to investigate the impact of shelter-in-place measures on human behavior, resting heart rate, sleep, and activity patterns in the context of telemedical services. We enrolled five adult volunteers of different age groups and backgrounds (three working participants and two octogenarians) who were smartwatch users and self-isolating at home during the Covid-19 pandemic. Eligible volunteers were those who met all of the following inclusion criteria for the study: 18 years of age or older, signed informed consent, stayed at home, and self-isolated during the lockdown period, and were Fitbit Versa users in the previous year. The last criterion was mandatory, as it was the only way to conduct a retrospective comparative analysis. Volunteers with dementia, stroke, and those who were not self-isolated at home were not eligible. The study protocol conformed to the guidelines set forth by the Declaration of Helsinki.

The self-isolate interval began on 10 March 2020 and ended on 30 April 2020 while the overall investigated interval began on 22 January 2019 and ended on 30 April 2020 (464 days).

To avoid bias in measurements among volunteers, we chose Fitbit Versa smartwatches, which allow for continuous recording of parameters: quality and duration of sleep, resting heart rate and the number of steps during the day. This choice was dictated by literature data which showed that the Fitbit Versa was 10 times more used in research projects and registered in ClinicalTrials than other brands [42].

Cardiovascular and Pre-Frailty Risk was assessed using a developed 4-point scale. The rationale for the selection of elements for the Cardiovascular and Pre-Frailty Risk Assessment is primarily the selection of available parameters measured with smartwatches and relating them to their suitability and usefulness for the elderly population, along with an appropriate justification of the selected parameters based on available literature data, cardiological (ESC) and geriatric guidelines and clinical experience. This scale could also be adapted in qualification for surgical procedures of the older adults. According to the ESC Guidelines (2016) [12], age is the dominant cardiovascular risk factor, but it should not be considered in isolation from other factors. Interestingly, Sergi et al. (2015) conducted a study with 1567 participants aged 65 to 96, which showed that pre-frailty is independently associated with a higher risk of developing cardiovascular disease in older adults [43]. The use of smartwatches to assess pre-frailty and cardiovascular risk is still unknown. We are the first to report the benefits of this type of measurement, especially among older adults. In our artificial intelligence-based approach to our Cardiovascular and Pre-Frailty Risk Assessment method, we mapped low physical activity (the component of frailty syndrome and cardiovascular risk) to fewer steps using the developed Python and Matlab scripts.

Aging is associated with a decreased ability not only to initiate but also to maintain sleep [44]. Deterioration of sleep in the older adults also correlates with deterioration of health and well-being. Sleep disturbances are very common in older patients with delirium. Delirium can be characterized by changes in the sleep-wake cycle and may be the first sign of deterioration in health, such as from infection [45,46,47].

In the Clinical point of view section, we emphasized the role of the resting heart rate as an important predictor of cardiovascular risk. Moreover, a recent study by Toosizadeh et al. (2021) suggested that measuring the dynamics of heart rate in response to daily activities could be a significant marker in screening for the frailty syndrome in older adults [48]. Our Cardiovascular and Pre-Frailty Risk Assessment method is a non-linear scale developed based on clinical practice. Reaching a high-risk state should be alarming and should prompt appropriate corrective action as it can still be reversible i.e., lifestyle change.

For validation we collected qualitative and narrative data from respondents using a proprietary questionnaire to verify the data collected by Fitbit Versa. The author’s qualitative questionnaire covering sociodemographic status, self-assessment of changes in behavior, mood, sleep, physical activity, and daily activities was conducted by researchers using videoconferencing at the end of the shelter-in-place.

### 3.3. Methods

The analysis was performed on the basis of qualitative research and the descriptive statistics. Overall, we created a dataset consisting of 464 days of continuous measurements that included 50 days of self-isolation at home during the Covid-19 pandemic. We used Fitbit Versa (dimensions: 1.98 × 3.98 × 9.00 inches) as a source of biomarkers, which is an advanced smartwatch featuring built-in wrist-based heart rate sensor, sleep tracker, accelerometer, 4 days battery life and Bluetooth wireless interface for Fitbit cloud communication. Data were extracted and analyzed from the Fitbit cloud application using developed Python and Matlab scripts. A quantitative and comparative study analysis was carried out using Python and Matlab.

The research was planned in such a way as to simultaneously compare changes in the measured physiological parameters, because the seasons have a large impact on the variability of human activity, e.g., in summer, human activity increases because it is warmer and there are more opportunities to spend time actively outdoors.

We believe that the comparison of physiological activity during hard lockdown with the same period in the preceding year is very reliable and reflects the real impact of the pandemic on human activity. A similar methodology was published by fitness tracker companies comparing the same periods to avoid confounding variables such as the seasons or holidays [49].

### 3.4. Statistical Analysis

For the purpose of statistical analysis, the study participants were divided into two groups: Group 1—professionally active participants (younger participants) and Group 2—octogenarians (participants aged ≥80 years). Descriptive statistics were based on the mean and standard deviation (SD). Student’s *t*-test was used to determine significant differences between the pre-Covid-19 and Covid-19 dataset. *p* < 0.05 were considered statistically significant. Statistical analysis was performed using the Statistica 13 program.

### 3.5. Definitions

Throughout the text, the authors use the following terms: self-isolation and shelter-in-place to denote the period of the official policy of hard national lockdown, which was introduced in Poland on 10th March 2020, due to the Covid-19 pandemic and meant a total ban on leaving home, except for the necessity to make necessary purchases, going to work, church, with a dog or taking out the rubbish. The complete lockdown policy expired after the restrictions were relaxed on 30 April 2020. All study participants were self-isolated at home during the follow-up period. All working participants (three subjects) worked remotely from home.

## 4. Results

We analyzed the experimental dataset using dedicated scripts developed in Python and Matlab from five subjects of varying age, sex, marital, and occupational status. All study participants performed restrictive self-isolation at home during the lockdown period and wore a Fitbit Versa device during the self-isolation period. No study-related adverse events were reported during the study. The study showed that the lockdown policy affected the level of activity of all study participants compared to the same period of the previous year, as well as the appropriate period before self-isolation. However, the impact was less among participants aged 80 and older, whose activity before sheltering was moderate, according to data obtained by Fitbit Versa. The most significant impact of social distancing was observed among young, active adults, whose activity dropped dramatically during the lockdown. The comparison of the resting heart rate, number of steps, and sleep duration in the analyzed periods was showed in Table 2. General characteristics were presented in Table 3.

### 4.1. Number of Daily Steps

**Figure 1:**
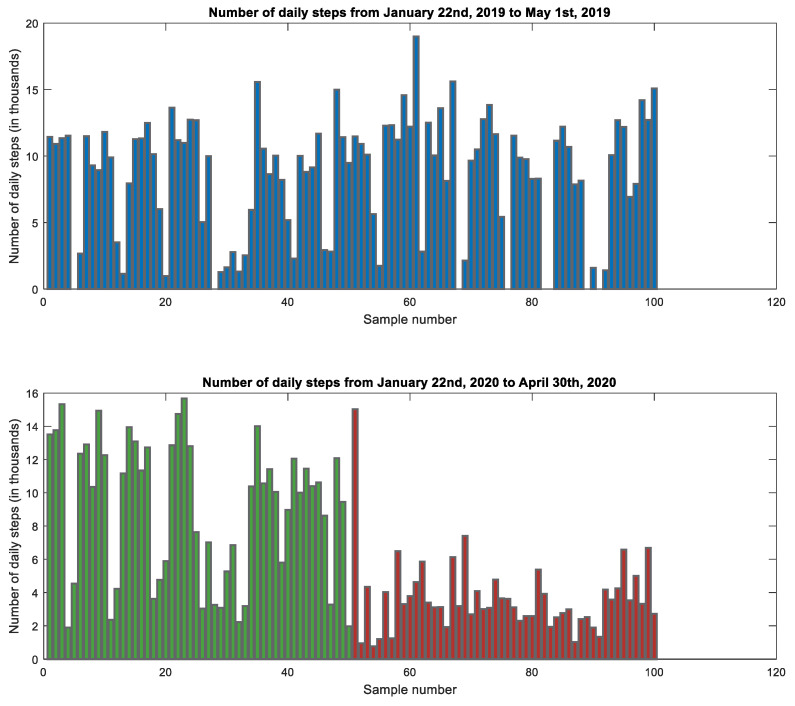
The number of daily steps in the analyzed intervals for Subject 1.

We compared the number of daily steps taken during the self-isolation period with the number of daily steps taken in the same period of the year before without self-isolation. A good comparison of subject 1 is shown in Figure 1. In this comparison, we found a significant decline in the number of daily steps during lockdown (red color) in comparison to the same period in the previous year (blue color). In addition, we observed a significant decline in the number of daily steps during the lockdown in comparison to the period of 50 days prior to isolation (green color). In both cases, it was observed that self-isolation significantly disrupted the mobility of all study participants based on their number of daily steps.

**Figure 2:**
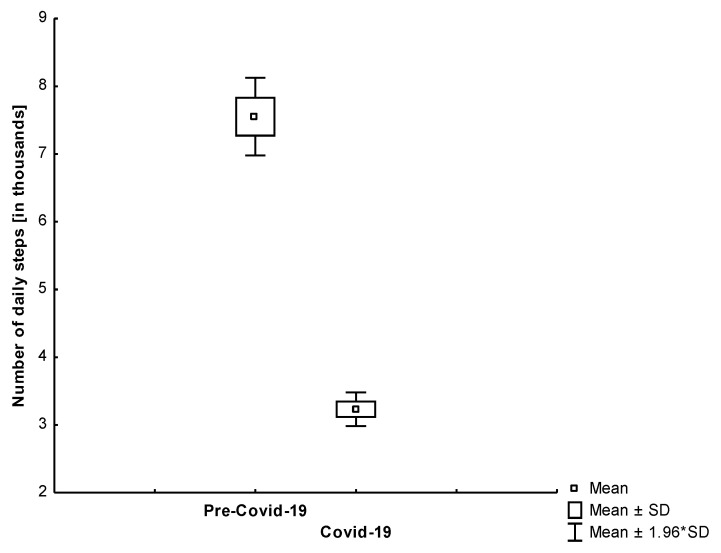
A comparison of the number of daily steps in the analyzed periods (*p* < 0.001).

**Figure 3:**
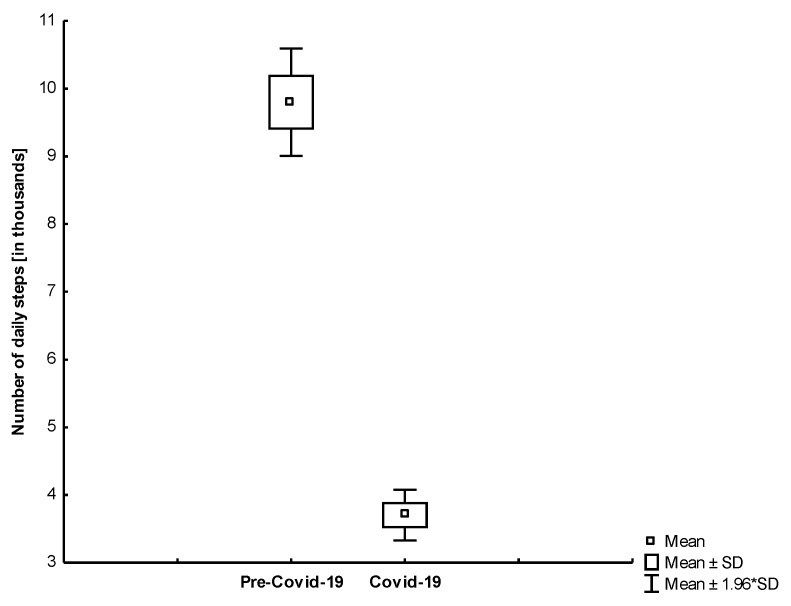
The comparison of the number of daily steps in the analyzed periods among young participants (*p* < 0.001).

**Figure 4:**
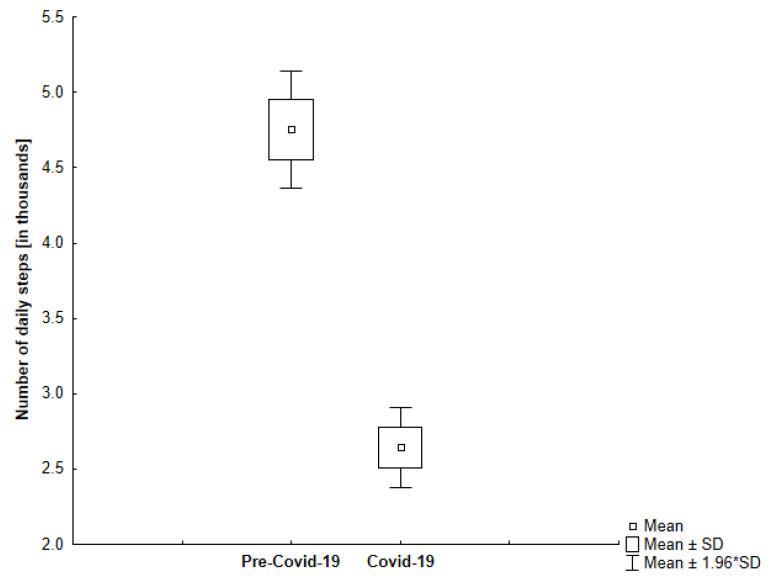
The comparison of the number of daily steps in the analyzed periods among older participants (*p* < 0.001).

As can be seen in Figure 2, the total number of daily steps during the self-isolation period was significantly lower than the total number of daily steps in the control measures performed by all study participants in the previous year. Figure 3 and Figure 4 show that both age groups had a significant (*p* < 0.001) decrease in the number of daily steps during the self-isolation period of Covid-19.

We found that in all study participants, the number of daily steps was lower in the self-isolation period compared to the time before sheltering in place (−57.21%; −4321; 95% CI, 3722 to 4920).

### 4.2. Resting Heart Rate

**Figure 5:**
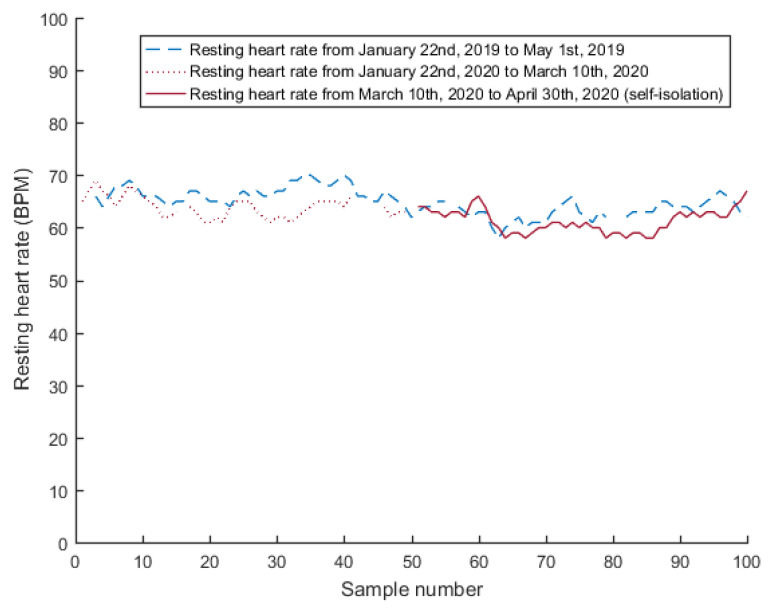
Resting heart rates (RHR) in the two analyzed intervals for Subject 4.

Resting heart rates (RHR) in the two analyzed intervals for Subject 4 are shown in Figure 5.

**Figure 6:**
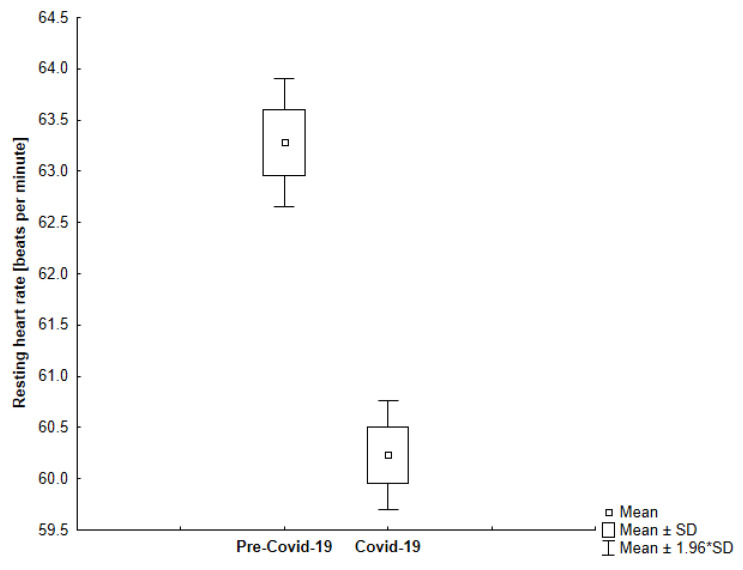
The comparison of the average resting heart rate in the analyzed periods (*p* < 0.001).

**Figure 7:**
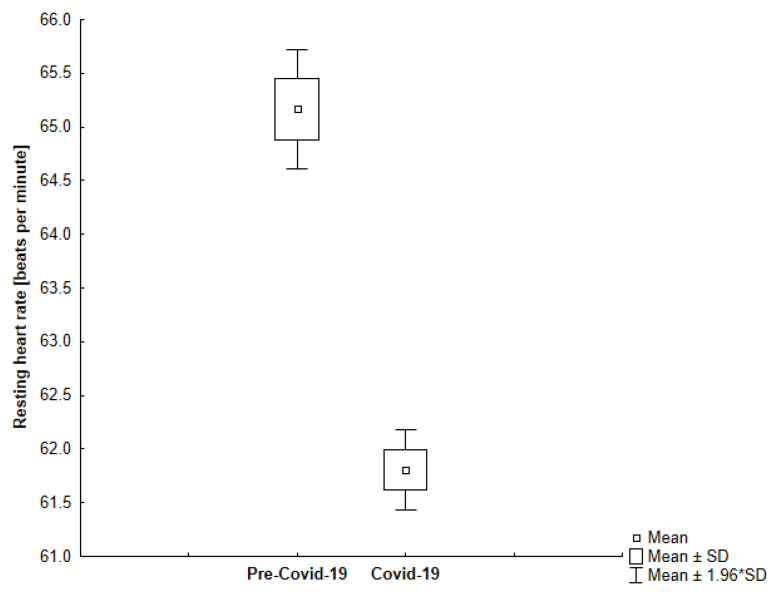
The comparison of the average resting heart rate in the analyzed periods among younger participants (*p* < 0.001).

**Figure 8:**
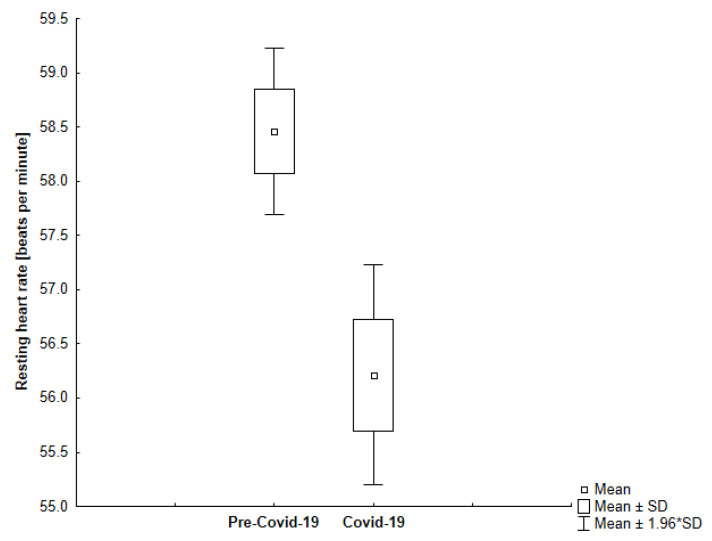
The comparison of the average resting heart rate in the analyzed periods among older participants (*p* < 0.001).

In our study, we observed that all participants in the study had a lower resting heart rate during self-isolation compared to the time before sheltering (−4.81%; −3.04; 95% CI, 2.59 to 3.51) which is shown in Figure 6. Interestingly, we found that the more active participants experienced a more significant decrease in their resting heart rate (Figure 7). However, we observed greater variability in resting heart rate in the elderly (Figure 8).

### 4.3. Sleep Duration and Quality

Our findings suggest that shelter-in-place measures a disrupted sleep duration. It turned out that the study participants went to bed later, and the results are challenging to interpret unequivocally.

**Figure 9:**
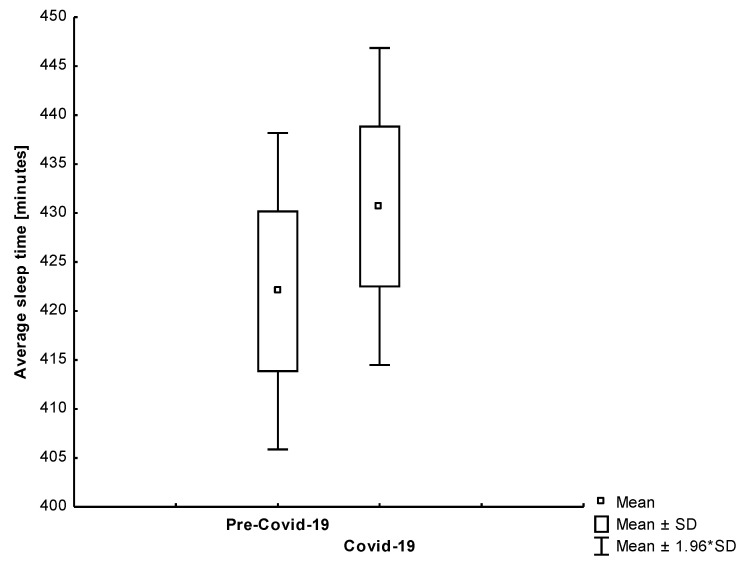
The comparison of the average sleep time in the analyzed periods among younger participants (*p* = 0.441).

**Figure 10:**
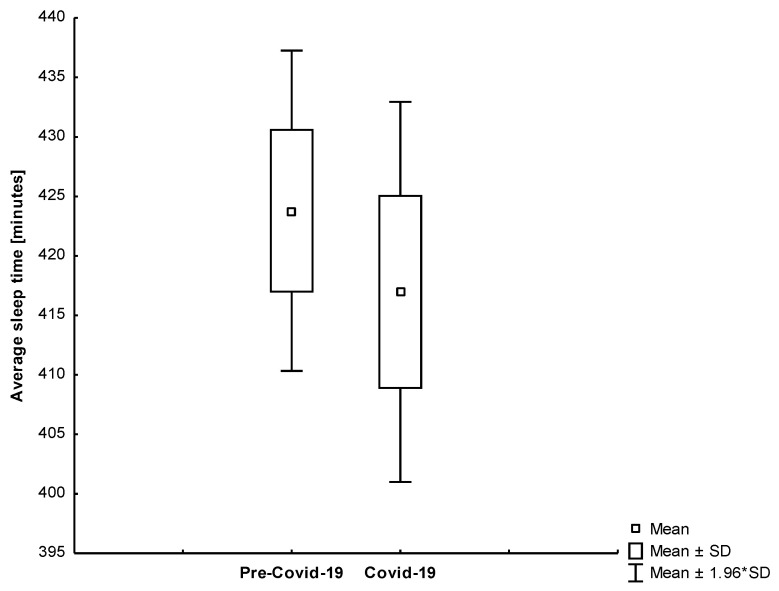
A comparison of the average sleep time between reference and self-isolation for all study participants (*p* = 0.499).

**Figure 11:**
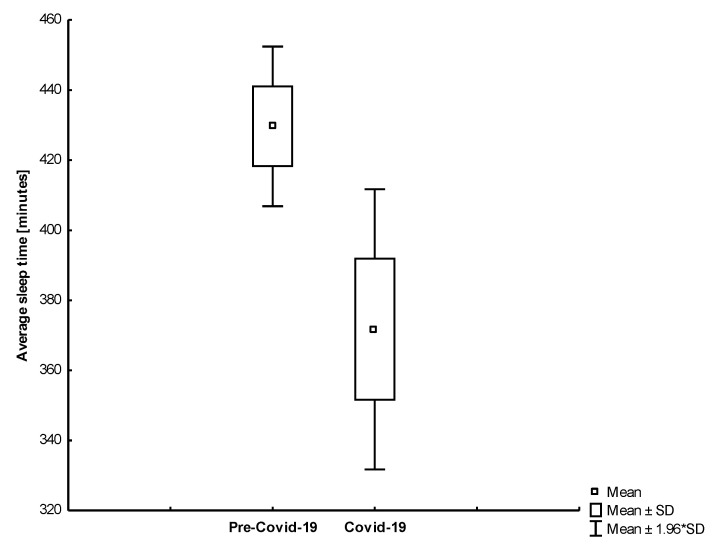
The comparison of the average sleep time in the analyzed periods among older participants (*p* = 0.008).

We also found that the results differed by age group. Conversely, we noticed a significant drop in the average sleep time among octogenarians (−13.48%; −57.91; 95% CI, 16.33 to 99.49). The rest of the study participants did not significantly benefit from self-isolation in terms of sleep duration. Only one study participant had the same duration of sleep in both periods. We found that younger and active participants increased the length of sleep (Figure 9). The most physically and professionally active subject from our study group (Subject 1) had a significant increase in sleep time during the lockdown. The comparison of the average sleep time between reference and self-isolation for all study participants was shown in Figure 10. Interestingly, we found that the length of sleep in octogenarians decreased significantly during the Covid-19 period (Figure 11).

### 4.4. Cardiovascular and Pre-Frailty Risk Assessment Scores

We proposed the non-linear method to quantify the Cardiovascular and Pre-Frailty Risk based on the following digital biomarkers: daily steps, resting heart rate, sleep duration, and age. The method is based on scoring each parameter on a 2-point scale. The scores 5.5 and above are considered increased cardiovascular and pre-frailty risk. A specialist geriatrician was asked to assess the increased cardiovascular and pre-frailty risk. Our findings suggest that shelter-in-place measures increased cardiovascular and pre-frailty risk scores in the majority of participants. Cardiovascular and Pre-Frailty Risk Scores of subjects were shown in Table 4.

## 5. Discussion

Over the past decade, we witnessed the development of wearable devices equipped with biometric sensors, such as smartwatches or fitness trackers, which are ubiquitous in the lives of active people who focus on fitness and exercise. Smartwatches are able to measure heart rate, falls or number of steps accurately. We believe that these parameters contain important diagnostic or screening information that can be used in clinical practice, in particular as a basis for teleconsultation with a primary care physician and may indicate an early stage of the disease.

### 5.1. Smartwatch

The Covid-19 pandemic and lockdown policies revealed how important a tool the smartwatch could be during sheltering to monitor people’s health and well-being. What is more, it could help doctors quickly diagnose and triage when healthcare systems are overwhelmed, and there is a shortage of medical personnel during a health crisis like the pandemic.

To the best of our knowledge, our study is the first to investigate the impact of a pandemic on activity levels measured using a smartwatch in the context of Cardiovascular and Pre-Frailty Risk Assessment. However, more research is needed to examine the safety and usefulness of this tool not only in diagnosing diseases but also in preventive strategies. The primary research contribution is information on how the complete lockdown at home affects the investigated parameters. Stay-at-home measures were shown be successful in limiting the spread of the infection, but it should not be forgotten that a sedentary lifestyle can lead to many cardiovascular complications [12]. Consequently, our results add to the state-of-the-art information about behavioral changes during a lockdown period and may help better support people by introducing an interactive exercise program in case of future lockdown policies.

Our findings reveal that self-isolation had an impact on health risk. Our results are consistent with the data provided by Fitbit, which conducted an analysis of the behavior of Fitbit users when shelter-in-place was ordered. The data showed that Fitbit users reacted to these policies and social distancing very quickly and thoughtfully, as a statistically significant decrease in the average number of steps was observed compared to the same period last year [49]. However, the severity of the decrease in the number of steps was found among European users, especially in Italy, Spain, Portugal, France, and Romania, in the range of a 7–38% decrease in the number of steps in the last week of March [49]. Interestingly, the largest decline was observed among users aged 18–29, with their steps between 16–23%, while users aged 65 and older had the lowest impact on activity levels (4–10%) [50]. A similar observation was also found in our study among octogenarians. This observation could be explained by the aging process and the tendency to decrease physical activity among older adults due to chronic diseases, frailty, sarcopenia, and disability [51]. Nowadays, the frailty syndrome in older adults—and its screening and diagnostic tools—are of great interest to many researchers [5].

Furthermore, it was observed that in locations with shelter-in-place mandates, the duration and quality of sleep shifted. People went to sleep later, but they had more sleep and quality rest. The quality of sleep was improved, which was visible in deep and REM sleep [52]. In contrast, in our study, we observed that most of the study participants experienced a significant reduction in sleep duration or did not improve sleep time during self-isolation, which could be due to poor sleep hygiene during the self-isolation period: lack of physical activity, eating or drinking before going to bed, working longer hours, or using a computer or smartphone before bedtime, which ultimately had a negative effect on sleep. Only one participant (subject 1) achieved the most significant benefit from the self-isolation period, which could be explained by fatigue and overwork before lockdown (it was the most active and working participant in the group). On the other hand, it could also be explained by the fact that Subject 1 regularly performed exercises during the self-isolation period, which had a positive impact on sleep. An interesting finding was also that most active people were affected to a greater extent by lockdown than those who were less active before. Octogenarians were less influenced by introduced measures because they had lower activity before. Our findings suggest that there was a significant reduction in the number of daily steps and resting heart rate for all study participants compared to the time before lockdown. We also believe that resting heart rate is an important indicator that plays a crucial role in managing anxiety and may be used as a metric for evaluating the stress level. This, apart from psychological surveys, can screen subjects with elevated stress levels or increased risk of medical condition e.g., fever.

Data provided by Fitbit demonstrated that social distancing and stay-at-home mandates affected all Fitbit users, but also proved that Fitbit users were actively involved in slowing the spread of the Covid-19. Contrary to the data published in the Fitbit study, in our study we carefully supervised study participants to ensure the proper study protocol and that our subject was completely isolated at home during the investigated period. However, in the Fitbit study, it was not possible to verify the quality of the data, as well as the correctness and coherence of the input data e.g., lack of information about wearing the device during all investigated period, obeying the lockdown, different lockdown dates and regulations in different countries, risk of sharing the device with family members, influence on results of unknown external factors, etc.

Our study had some limitations. The major limitation is the fact that our findings are based on a relatively small sample size. However, our study is rather a case study and could be an example of a restrictive lockdown policy, which was verified by researchers. What is more, our results are consistent with the results that were collectively presented by Fitbit globally and in different countries separately. Another limitation may be the selection of the time window for comparing lockdown state. The main limitation of our study was that researchers were unable to thoroughly verify that the participant was 100% adhering to the lockdown period. The developed pre-frailty risk assessment method is limited to five biomarkers and may be improved in the future if more health data can be harvested from the smartwatch i.e., glucose levels etc.

### 5.2. Telemedical Solutions during the Covid-19

Despite the many telemedical options available on the market that could offload hospitals and medical staff during the Covid-19 pandemic crisis, which dramatically hit many healthcare systems, especially in Italy, causing more than 24,000 deaths, there was no advocacy for using available telemedicine to provide continuity of care solutions for the chronically ill [39]. The only telemedical method used was a smartphone application for contact tracing. However, in a lockdown situation, an increase in the use of home telemonitoring systems to monitor vital signs was observed, which helped about half of the patients with chronic diseases to adjust the appropriate treatment [53]. Implemented remote patient-monitoring initiatives, such as Telehealth Intervention Programs for Seniors, that provide a weekly assessment of vital signs for low-income older people, can be an example of a successful program that reduces the number of hospital visits among older people [54].

One of the interesting telemedical solutions used in China during the Covid-19 pandemic was a mobile telehealth system, which was used to facilitate the presentation and discussion on the case study. This could help prevent the mobility of doctors in the hospital for consultation and decrease person-to-person contact to reduce the risk of transmission of infection between healthcare professionals [55].

According to the literature, the smartphone can be used as an adaptive physical activity smartphone intervention using a unique application supporting its positive effect on physical activity and a sedentary lifestyle [56]. However, during a pandemic and lockdown policy, smartphones do not seem to be such a useful tool to monitor the level of human activity because users do not wear them all the time at home.

The Covid-19 pandemic also had a significant impact on mental health. Isolation, social distancing, job loss, workload, and high exposure to coronavirus among healthcare professionals could lead to psychological distress and many adverse effects on mental health, including depressive symptoms, anxiety, burnout, and exacerbation of mental illness [57]. Telemental health services such as counseling, supervision, training, and psychoeducation via online platforms could provide adequate support to patients, family members, and health service providers during a pandemic [57].

### 5.3. The Post-Covid-19 Face of Telemedical Services

The pandemic redefined the use of primary care, healthcare services and the physical examination process. In the absence of data from physical examinations, the only source of information about the general health of the patient were smartwatches and home medical devices. We hypothesize that a novel category of medical devices is being introduced—a smartwatch or medical wearable device (certified in accordance with Regulation (EU) 2017/745 of the European Parliament and of the Council of 5 April 2017 on medical devices, PN-EN ISO 13485:2016 standards), which will focus on recording overall health on the basis of available data, which can be accurately recorded by wearable device sensors (e.g., smartwatch). These categories of devices may redefine the way primary care is delivered because physical examination can progress to remote examination, supported by data from a certified wearable medical device, which can improve diagnostic decisions and screening. The smartwatch already has the ability to synchronize health data with the cloud service, which allows easy remote sharing of health data with a primary care physician without additional cloud infrastructure. This allows for fast deployment of telemedical services based on wearable devices and scalability of solutions. Evaluation of the clinical effectiveness of wearable medical devices and mindset change of both healthcare professionals and patients are among important challenges in the adoption of wearable telemedical services. However, consideration should be given to ensuring the confidentiality, integrity, and security of sensitive medical data so as to prevent access by unauthorized persons. Other challenges include ensuring interoperability and supervision over the place where medical data is stored, along with compliance with related regulations and standards. Furthermore, there are wearable devices that are certified as medical devices and are FDA-approved for certain health indications, such as the Apple Watch and the Omron Health Guide Watch [58].

## 6. Conclusions

We presented a developed and validated methodology that we used to assess cardiovascular and pre-frailty risk during the Covid-19 shelter-in-place measures on human activity patterns based on multimodal biomarkers collected from smartwatch sensors. The developed method can be easily deployed in the software update of the smartwatches or other wearable devices and might be a useful health screening metric. We believe that in the future, smartwatches or novel wearable medical devices may play an essential role in telemedical services, especially during emergency scenarios or health crisis. However, the impact of long isolation on health is unknown and will be the subject of future research. Future research will also focus on human aspects during shelter-in-place measures. Our findings may be useful to policymakers and can be used to find solutions to support people, especially older adults, during the lockdown period and improve the quality of telemedical services.

## Figures and Tables

**Table 1:** Cardiovascular and Pre-Frailty Risk Assessment method.

Biomarker	Scores					Assumptions and References
	0	0.5	1.0	1.5	2.0	
**Daily Steps**	>12,500	10,000–12,500	7500–9999	5000–7499	<5000	[38,39]
**Resting Heart Rate**	<60 bpm	60–67 bpm	68–74 bpm	75–80 bpm	>80 bpm	[21,22,23,40]
**Sleep Duration**	7–8 h/day	-	-	-	<7 h/day or >8 h/day	[27,28,29]
**Age**	<70	70–74	75–79	80–84	85+	[41]

**Table legend:** bpm—beats per minute.

**Table 2:** The comparison of the resting heart rate, number of daily steps, sleep duration in the analyzed periods.

Variable		Pre-Covid-19		Covid-19		*p*-Value
		Mean	±SD	Mean	±SD	
**Resting Heart Rate [bpm]**	All	63.28	4.36	60.23	3.69	<0.001
	Group 1	65.17	3.28	61.80	2.18	<0.001
	Group 2	58.46	2.81	56.21	3.73	<0.001
**Steps Count [results in thousands]**	All	7550	4430	3230	1910	<0.001
	Group 1	9800	4570	3700	2160	<0.001
	Group 2	4750	2010	2640	1360	<0.001
**Sleep Duration [min.]**	All	423.79	81.84	416.97	97.12	0.499
	Group 1	422.02	86.07	430.67	86.21	0.441
	Group 2	429.64	66.80	371.73	117.19	0.008

**Table legend:** All—all participants, Group 1—professionally active participants (younger participants), Group 2—octogenarians (older participants), bpm—beats per minute, min—minutes, SD—standard deviation.

**Table 3:** General characteristics of the study participants.

Characteristic	Subject ID 1	Subject ID 2	Subject ID 3	Subject ID 4	Subject ID 5
Age	32	82	84	34	57
Gender	Female	Female	Male	Male	Female
Race	White	White	White	White	White
Marital Status	Single	Married	Married	Single	Divorced
Occupational Status	Employment	Pension	Pension	Employment	Employment
Number of Household Members	1	3	3	1	3
Current Smoker	No	No	No	No	No
Number of Diagnosed Chronic Diseases	0	8	9	1	1
Number of Chronic Medication Used	0	10	11	1	2
History of Cardiovascular Diseases:					
Hypertension	-	+	+	-	-
Chronic Heart Failure	-	+	+	-	-
Coronary Heart Disease	-	+	+	-	-
Atrial Fibrillation	-	-	+	-	-
Dyslipidemia	-	+	+	-	-
Type 2 Diabetes	-	+	-	-	-
Thyroid Disease	-	+	-	+	-
Functional Status:					
Uses Assistive Device for Ambulation	-	-	-	-	-
Able to Ambulate without Assistive Device	+	+	+	+	+

Table legend: + (yes), - (no).

**Table 4:** Cardiovascular and Pre-Frailty Risk Assessment (reference versus self-isolation).

Biomarker	Subject ID 1		Subject ID 2		Subject ID 3		Subject ID 4		Subject ID 5	
	Reference	Self-Isolation	Reference	Self-Isolation	Reference	Self-Isolation	Reference	Self-Isolation	Reference	Self-Isolation
Daily steps	0.5	2	2	2	2	2	0.5	2	0.5	2
Resting heart rate	1	0.5	0	0	0.5	0.5	0.5	0.5	0.5	0.5
Sleep duration	0	0	2	2	0	2	0	2	0	0
Age	0	0	1.50	1.50	2	2	0	0	0	0
Cardiovascular and Pre-Frailty Risk score	1.5	2.5	5.5	5.5	4.5	6.5	1	4.5	1	2.5
Clinical validation–Cardiovascular and Pre-Frailty Risk	N	N	**Y**	**Y**	N	**Y**	N	N	N	N

**Legend:** N—no, Y—yes.

## Data Availability

The data presented in this study are available on request from the corresponding author.
